# Supplementary material for: Tumor suppressor p53 regulates intestinal type 2 immunity
Source: Nat Commun. 2021 Jun 7;12:3371. doi: 10.1038/s41467-021-23587-x (PMC8184793; doi:10.1038/s41467-021-23587-x)
Supplement: Supplementary file 1 — Supplementary Information [file 41467_2021_23587_MOESM1_ESM.pdf]

Supplementary Figures

Supplementary Figure 1

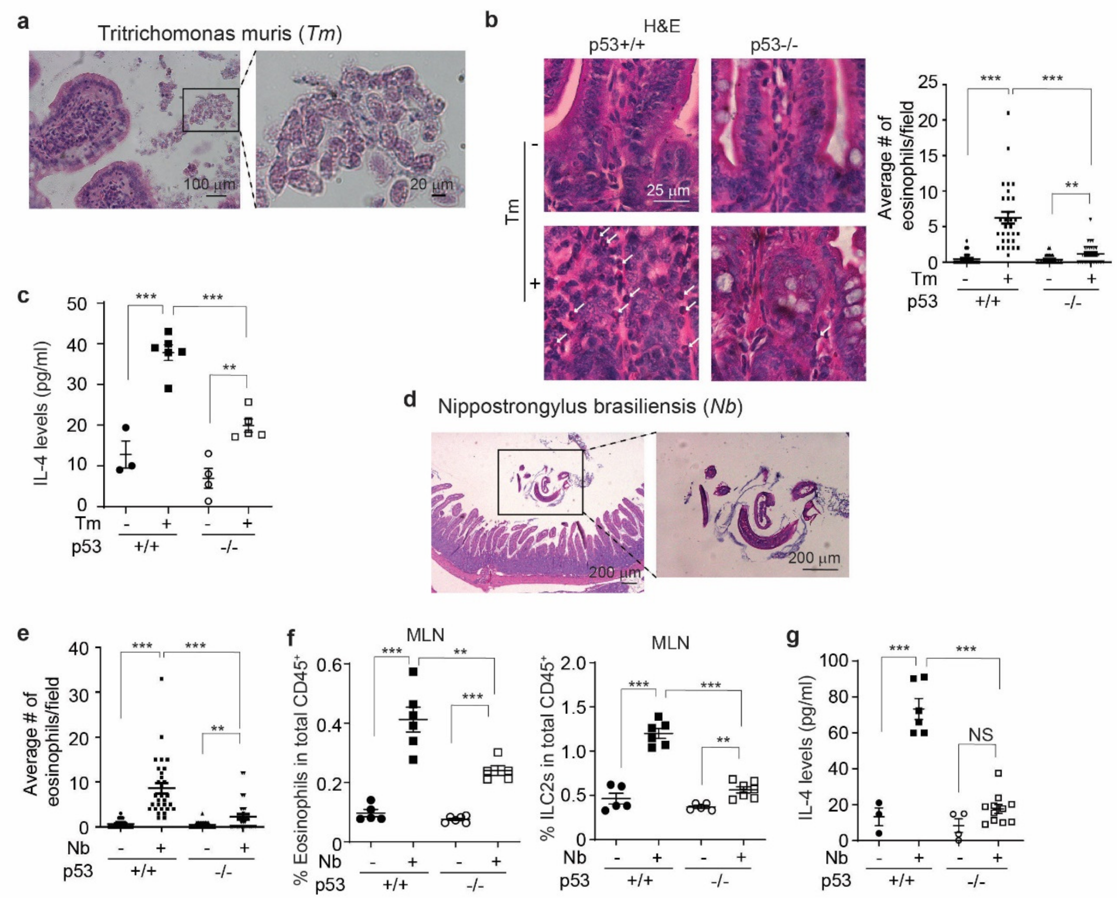

Supplementary Figure 1. The type 2 innate immune response towards parasitic infections in *p53+/+* and *p53-/-* mice. a. H&E staining of the small intestine of *Tm*-infected *p53+/+* mice at 21 d.p.i. showing the presence of *Tm*. **b**. *Tm* infection increased the population of eosinophils in the small intestine of both *p53+/+* and *p53-/-* mice, but the effect on p53-/- mice was markedly lower. Left panels: representative H&E images. White arrows indicate eosinophils. Right panel: quantifications of the number of eosinophils/field under 400x magnification. **c.** The levels of serum IL-4 in naïve and *Tm*-infected *p53+/+* and *p53-/-* mice determined by ELISA assays. **d.** H&E staining of the small intestine of *Nb*-infected *p53-/-* mice at 7 d.p.i. showing the presence of *Nb* worms. **e**. Quantifications of the number of eosinophils in the small intestine of naïve and *Nb*-infected *p53+/+* and *p53-/-* mice. **f**. Flow cytometric analysis of the population of eosinophils (left) and ILC2s (right) in MLNs from naïve and *Nb*-infected *p53+/+* and *p53-/-* mice. **g**. The levels of serum IL-4 in naïve and *Nb*-infected *p53+/+* and *p53-/-* mice. For **b**, **c**, **e**, **f** & **g**, data are presented as mean ± SEM. For **b** & **e**, n=30 fields from at least 3 mice/group. For **c**, **f** & **g**, each dot represents a mouse. For **c** & **g**, n=3-4/group for naïve mice and n=5-11/group for infected mice. For **f**, n=5-7/group. **: p<0.01; ***: p<0.001; NS: non-significant, two-tailed Student's *t*-test.

Supplementary Figure 2

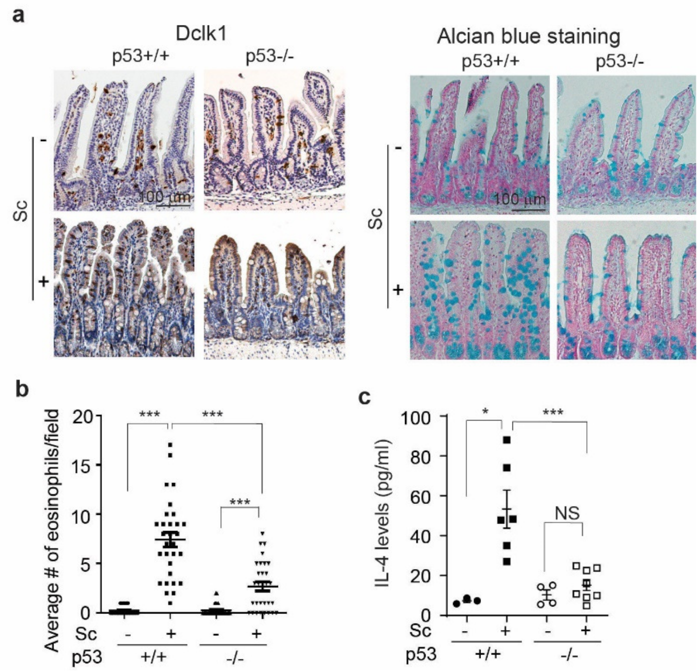

Supplementary Figure 2. p53 deficiency impairs type 2 immune response towards succinate treatment. *p53+/+* and *p53-/-* mice were given drinking water with or without 150 mM succinate (Sc) for 7 days. **a**. Representative images of Dclk1 IHC staining to detect tuft cell expansion (left panels) and Alcian blue staining to detect goblet cell hyperplasia (right panels) in naïve and Sc-treated *p53+/+* and *p53-/-* mice. **b**. Quantifications of the number of eosinophils detected by H&E staining in the small intestine of naïve and Sc-treated mice. n=30 fields from at least 3 mice/group. **c**. The levels of serum IL-4 in naïve and Sc-treated mice determined by ELISA assays. n=3-4/group for naïve mice and n=6-8/group for Sc-treated mice. Data are presented as mean ± SEM. *: p<0.05; ***: p<0.001; NS: non-significant, two-tailed Student's t-test.

Supplementary Figure 3

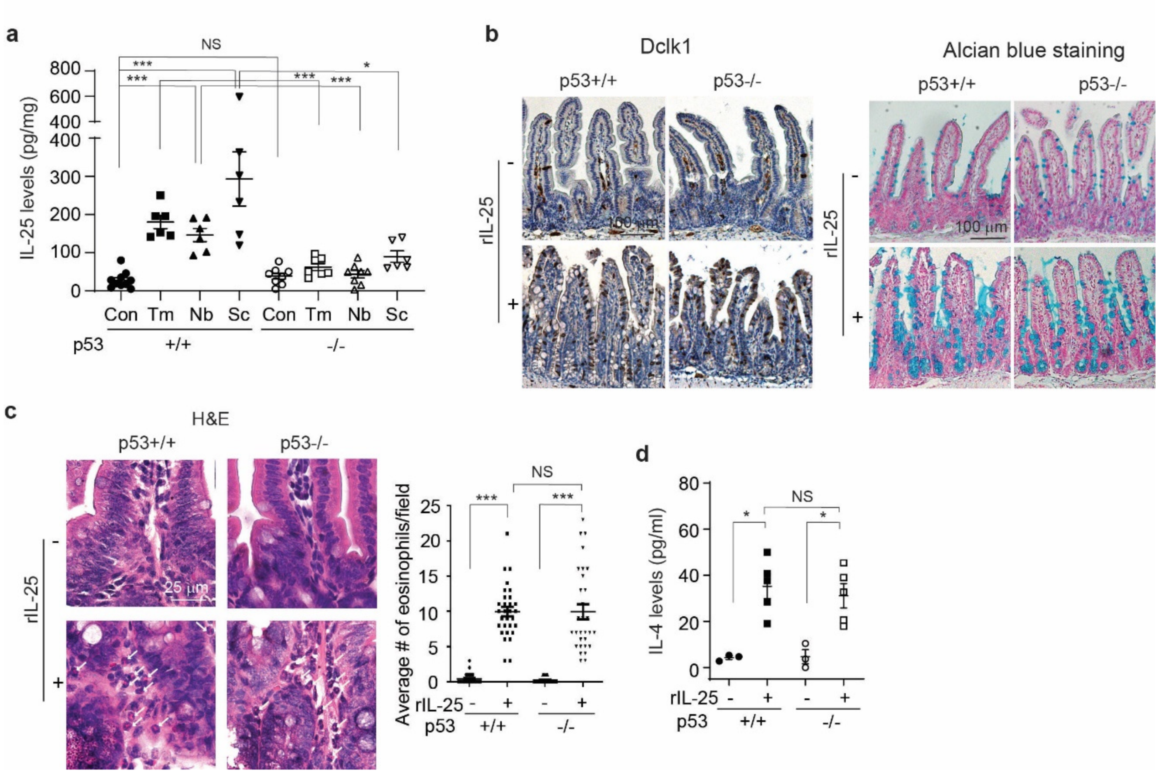

Supplementary Figure 3. Administering rIL-25 activates the type 2 immune response in both *p53+/+* and *p53-/-* mice. **a.** IL-25 levels in the small intestine of naïve mice, mice infected with *Tm* or *Nb*, and mice treated with succinate determined by ELISA assays. n=6-10 mice/group. **b-d.** rIL-25 administration restored the type 2 immune response in *p53-/-* mice. *p53+/+* and *p53-/-* mice were treated with rIL-25 (*i.p.*;0.5µg/day) or PBS for 7 days. **b**. Representative images of Dclk1 IHC staining detecting tuft cell expansion (left) and Alcian blue staining detecting goblet cell hyperplasia (right) in naïve and rIL-25-treated mice. **c**. Quantifications of the population of eosinophils detected by H&E staining in the small intestine in naïve and rIL-25-treated mice. Left panels: representative H&E images. White arrows indicate eosinophils. Right panel: quantifications of the number of eosinophils/field under 400x magnification. n=30 fields from at least 3 mice/group. **d**. The serum IL-4 levels in naïve and rIL-25-treated mice determined by ELISA assays. n=3/group for naïve mice and n=5/group for rIL-25-treated mice. Data are presented as mean ± SEM. *: p<0.05; ***: p<0.001; NS: non-significant, two-tailed Student's t-test.

Supplementary Figure 4

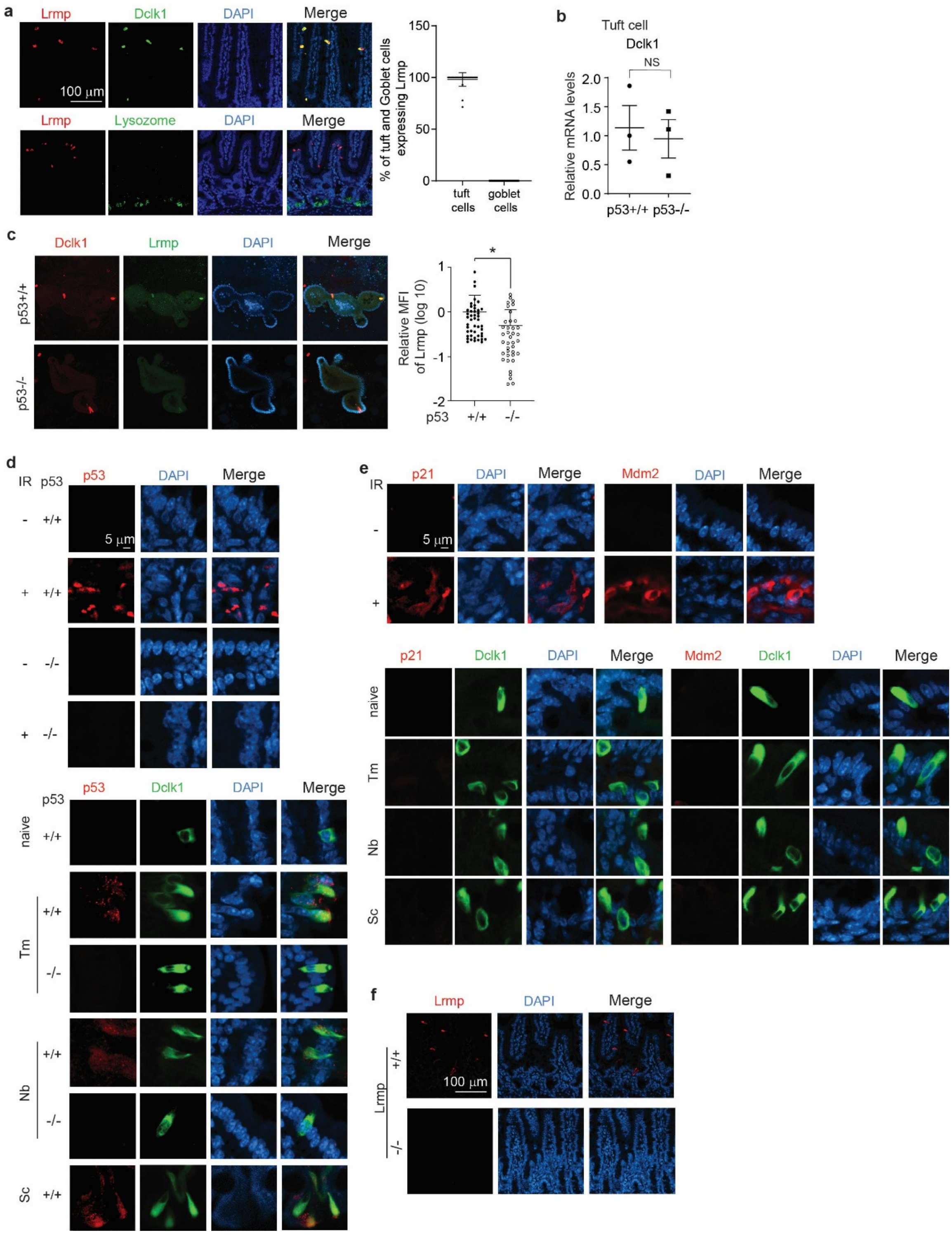

Supplementary Figure 4. Lrmp is specially expressed at higher levels in tuft cells and p53 upregulates Lrmp levels in intestinal tuft cells. a. Lrmp expression in tuft cells and paneth cells of mouse intestinal tissues determined by IF staining using anti-Lrmp

antibodies and either anti-Dclk1 (for tuft cells) or anti-lysozome (for paneth cells) antibodies. **b.** Relative mRNA levels of Dclk1 in intestinal tuft cells of naïve *p53+/+* and *p53-/-* mice determined by quantitative RT-PCR and normalized with β-actin. Tuft cells were enriched from the small intestine as $EpCAM^+$ Siglec-F^+ cells. n=3 mice/group. **c.** The lower Lrmp levels in the tuft cells of *p53-/-* intestinal organoids than *p53+/+* intestinal organoids as determined by IF staining. Left panels: Representative IF staining images. Right panel: Quantifications of mean florescence intensity (MFI) of Lrmp in tuft cells of *p53+/+* and *p53-/-* intestinal organoids. **d** & **e.** p53 (**d**), p21 and MDM2 (**e**) protein levels in the intestinal tissues from mice with γ-irradiation, *Tm* and *Nb* infections, or succinate treatment as determined by IF staining. **f.** Lrmp expression in the intestinal tissues of *Lrmp+/+* and *Lrmp-/-* mice determined by IF staining using anti-Lrmp antibodies. Data are presented as mean ± SD. *: $p<0.05$; NS: non-significant, two-tailed Student's *t*-test.

Supplementary Figure 5

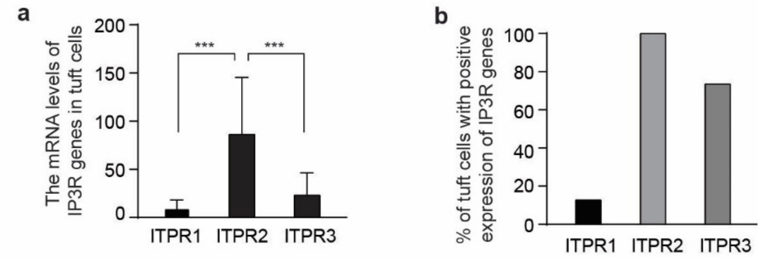

Supplementary Figure 5. The expression of IP3R genes in mouse intestinal tuft cells by analyzing a scRNA-seq dataset (GSE92332). a. The mRNA levels of IP3R genes in tuft cells. **b**. The percentage of tuft cells with positive expression of IP3R genes. Total of 102 tuft cells were sequenced in this dataset. ***: p<0.001, two-tailed Student's t-test.

Supplementary Figure 6

a

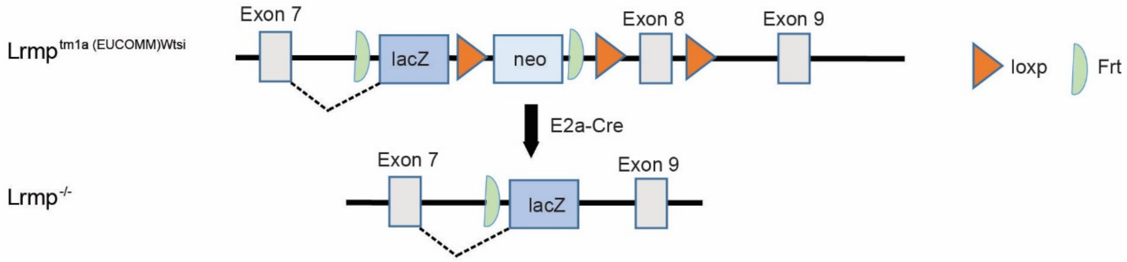

b

		Primer sequences	Product size
WT	Forward	5'-AGCACAGAGCGCTTTGGATATGAAG-3'	169bp
	Reverse	5'-CACCGGAACTGGCACTTACTCTGG-3'	
Lrmp-/-	Forward	5'-CAGAGGAAGGCAGGAGCTGTTTTGAG-3'	420bp
	Reverse	5'-CGCTACCATTACCAGTTGGTCTGGTG-3'	

c

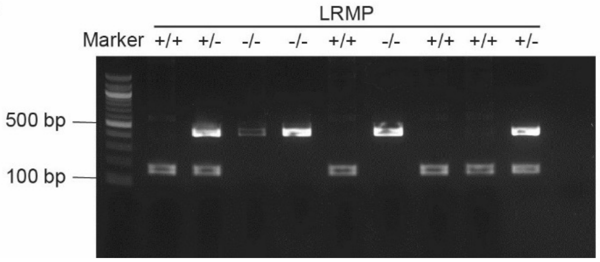

Supplementary Figure 6. Generation and characterization of *Lrmp-/-* mice. a. Schematic representation of the strategy to generate *Lrmp-/-* mice. **b**. Primers used for PCR genotyping analysis. **c**. PCR genotyping analysis of *Lrmp*+/+, +/- and -/- mice.

Supplementary Figure 7

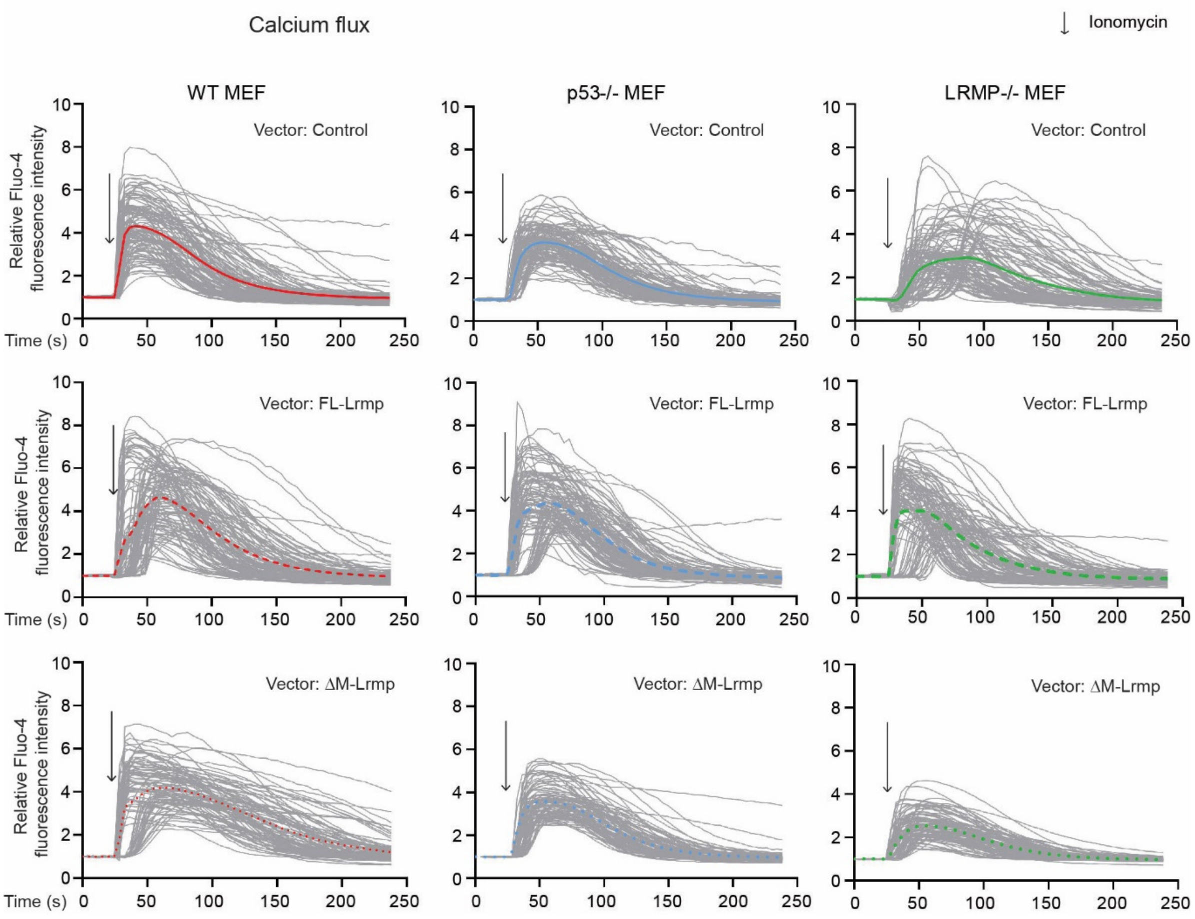

Supplementary Figure 7. Traces of Ca^{2+} flux in response to ionomycin treatment in WT, *p53-/-*, and *Lrmp-/-* MEFs. Each gray line represents the trace of Ca^{2+} flux of an individual cell in response to ionomycin treatment measured by using Fluo-4 AM with a time-lapse confocal microscope. At least 90 cells were analyzed in each group. The colored line represents the average of the cell population.

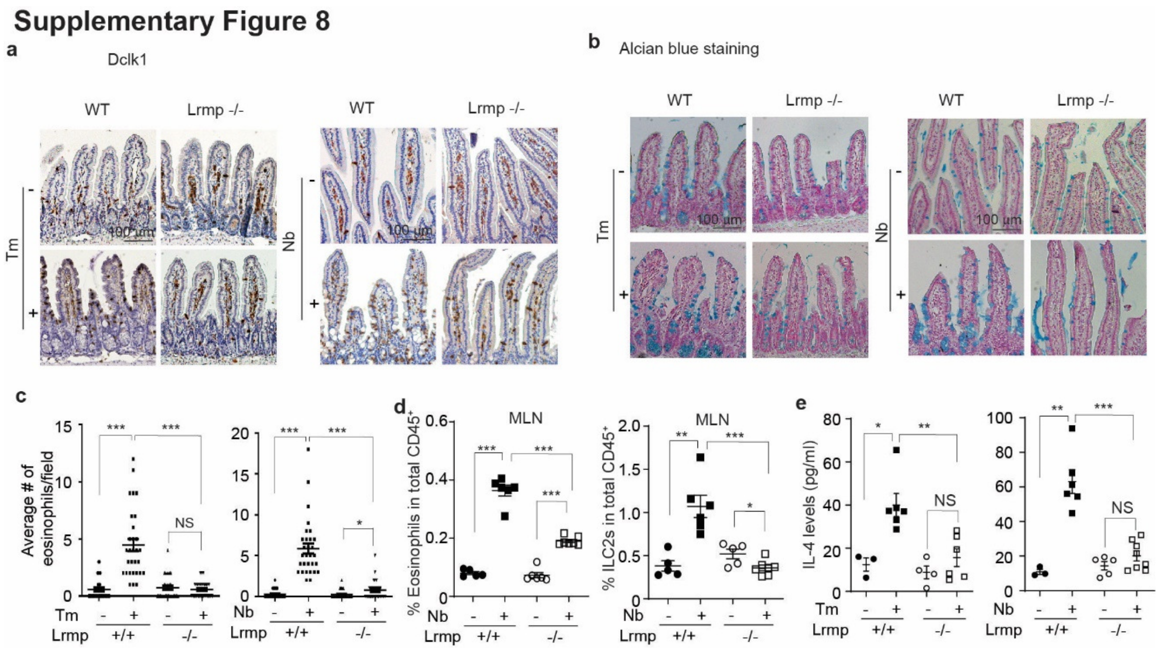

Supplementary Figure 8. The type 2 immune response towards parasitic infections is impaired in *Lrmp-/-* mice. WT and *Lrmp-/-* mice were infected with *Tm* or *Nb* and examined at 21 d.p.i. (for *Tm*) and 5 or 7 d.p.i. (for *Nb*), respectively. **a** & **b.** Representative images of Dclk1 IHC staining to detect tuft cells (**a**), and of Alcian blue staining to detect goblet cells (**b**) in the small intestine of naïve or infected mice. **c.** Quantifications of the number of eosinophils detected by H&E staining in the small intestine of naïve or infected mice. n=30 fields from at least 3 mice/group. **d**. Flow cytometric analysis of the population of eosinophils (left) and ILC-2 cells (right) in MLNs from mice with or without *Nb* infection. n=5-6 mice/group. **e**. The levels of serum IL-4 in naïve or infected mice determined by ELISA assays. n=3 mice/group for naïve mice and n=6-8 mice/group for infected mice. Data are presented as mean ± SEM. For **d**-**e**, each dot represents an individual mouse. *: $p<0.05$; **: $p<0.01$; ***: $p<0.001$; NS: non-significant, two-tailed Student's *t*-test.

Supplementary Figure 9

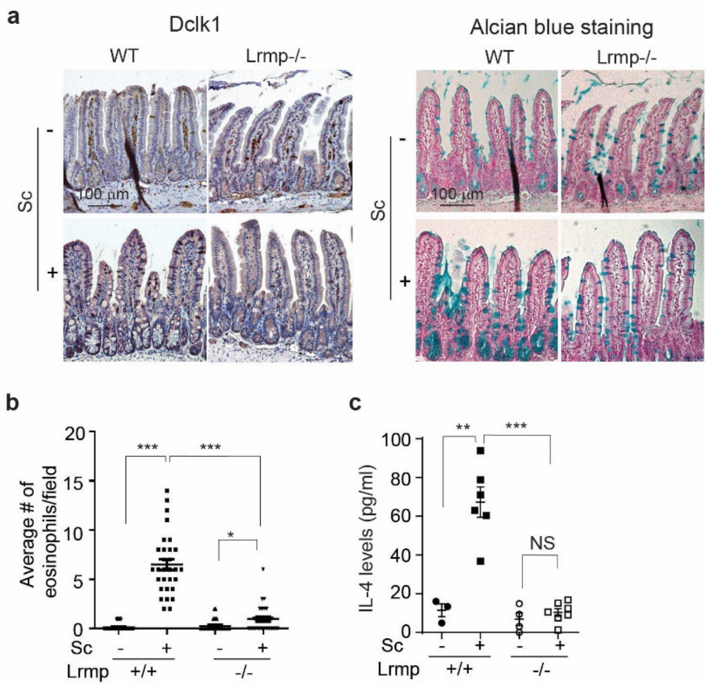

Supplementary Figure 9. The impaired type 2 immune response towards succinate treatment in *Lrmp-/-* mice. WT and *Lrmp-/-* mice were provided with drinking water with or without 150 mM succinate (Sc) for 7 days. **a**. Representative images of Dclk1 IHC staining (left panels) and Alcian blue staining (right panels) to detect tuft cells and goblet cells, respectively, in mice with or without Sc treatment. **b**. Quantifications of the number of eosinophils detected by H&E staining in the small intestine of mice with or without Sc treatment. n=30 fields from at least 3 mice/group. **c**. The levels of serum IL-4 in mice with or without Sc treatment determined by ELISA assays. n=3 mice/group for naïve mice and n=6-7 mice/group for Sc-treated mice. Data are presented as mean ± SEM. *: $p<0.05$; **: $p<0.01$; ***: $p<0.001$; NS: non-significant, two-tailed Student's *t*-test.

Supplementary Figure 10

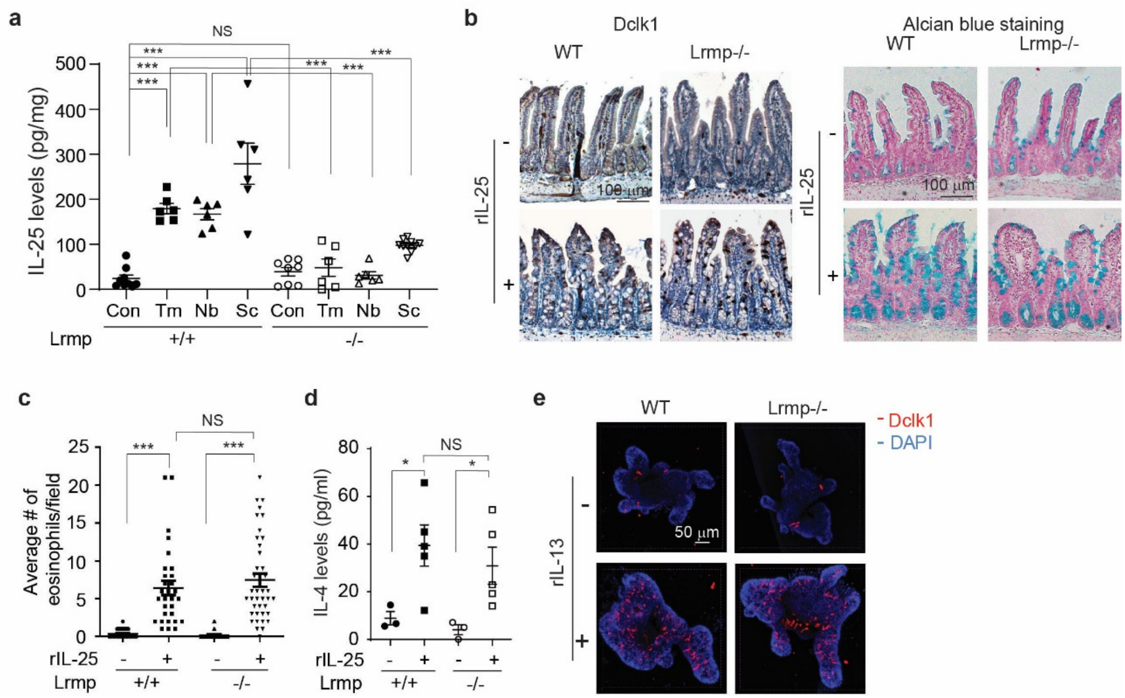

Supplementary Figure 10. Administering rIL-25 activates the type 2 immunity in *Lrmp-/-* mice. a. IL-25 levels in the small intestine of naïve mice, mice infected with *Tm* or *Nb*, and mice treated with succinate determined by ELISA assays. n=6-10 mice/group. **b-d.** WT and *Lrmp-/-* mice were treated with rIL-25 (*i.p.*; 0.5 µg/day) or PBS for 7 days. **b**. Representative images of Dclk1 IHC staining (left panels) and Alcian blue staining (right panels in mice with or without rIL-25 treatment. **c**. Quantifications of the number of eosinophils detected by H&E staining in the small intestine of mice with or without rIL-25 treatment. n=30 fields from at least 3 mice/group. **d**. The levels of serum IL-4 in mice with or without rIL-25 treatment determined by ELISA assays. n=3/group for naïve mice and n=5/group for rIL-25-treated mice. **e.** Recombinant IL-13 (rIL-13; 10 ng/ml for 48 h) induced tuft cell expansion to a similar extent in WT and *Lrmp-/-* intestinal organoids. Tuft cells in intestinal organoids were detected by IF staining of Dclk1. For **a**, **c** & **d**, data are presented as mean ± SEM. *: p<0.05; ***: p<0.001; NS: non-significant, two-tailed Student's t-test.

Supplementary Figure 11

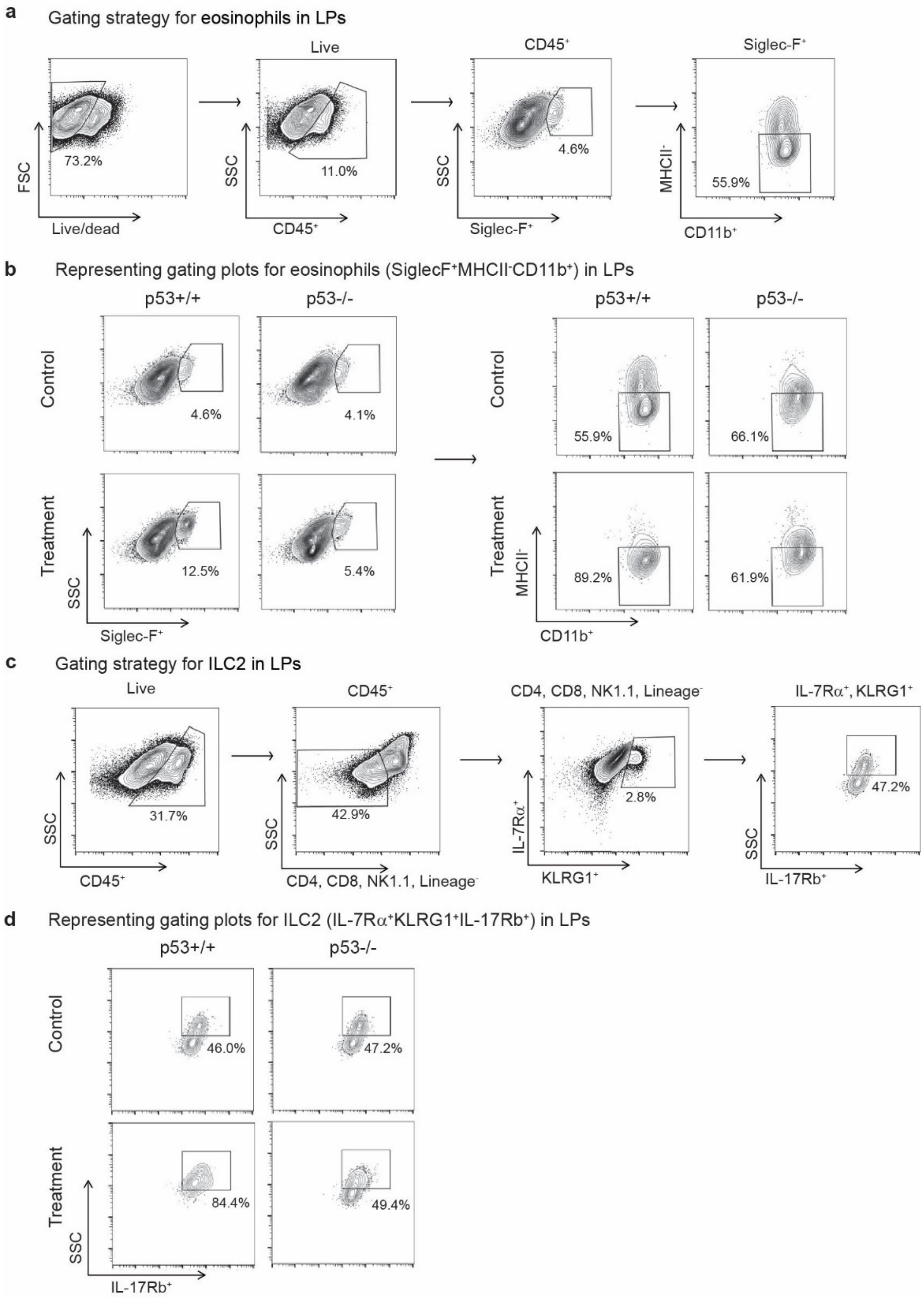

Supplementary Figure 11. Gating strategies and representative flow cytometry plots to detect eosinophils and ILC2s. **a** & **b**. Gating strategies (**a**) and representative flow plots (**b**) for eosinophils in LPs. **c** & **d**. Gating strategies (**c**) and representative flow plots (**d**) for ILC2s in LPs. A p53+/+ control sample and a p53-/- control sample were used to present gating strategies for eosinophils and ILC2s in LPs, respectively.

Supplementary Tables

Supplementary Table 1. The sequences of primers used in this study

Target	Sequences (5'-3')
For ChIP assays and Luciferase reporter assays	
Site 1	Forward: CGGGGTACCGCAGGTGTAGGTGAGATAAGCAGATGATG
	Reverse: CCGCTCGAGGGAGGAGGACCCAGGCACAGAC
Site 2	Forward: CCGGGTACCATGGAAAATATGGAACTCGCTACTGCTC
	Reverse: CCGCTCGAGACTCTTGCTTGTTTGTGTTTGTTTTGTTTC
For Lrmp vector cloning	
FL	Forward: GGGGTACCATGCTCTGTGTAAAAGGTCCCCCAGA
	Reverse: CGGGATCCCTACACTGGCAGTGGTCCATCATGC
ΔN	Forward: ATCGAATTCGCCACCATGAGGTTATCTTTGGGACTTAA
	Reverse: ATCGGATCCCACTGGCAGTGGTCCATCAT
ΔM	Forward 1: GGGGTACCATGCTCTGTGTAAAAGGTCCCCCAGA
	Reverse 1: GCTTTTTAATGAGGAACTCTGCCTCCAC
	Forward 2: AGAGTTCCTCATTAAAAAGCGTTCATCTTCTC
	Reverse 2: CGGGATCCCTACACTGGCAGTGGTCCATCATGC
ΔC1	Forward: GGGGTACCATGCTCTGTGTAAAAGGTCCCCCAGA
	Reverse: CGGGATCCCTAGCGGTGCTCACCCTGCTTTG
ΔC2	Forward: GGGGTACCATGCTCTGTGTAAAAGGTCCCCCAGA
	Reverse: CGGGATCCCTAGCAGATTGTGCCCCTGTCCC
ΔC3	Forward 1: GGGGTACCATGCTCTGTGTAAAAGGTCCCCCAGA
	Reverse 1: CCACTGAGCTCTGACAGTCATCTTCATCT
	Forward 2: TGACTGTCAGAGCTCAGTGGCTTCCT
	Reverse 2: CGGGATCCCTACACTGGCAGTGGTCCATCATGC
For qRT-PCR assays	
Actin	Forward: GAACCCTAAGGCCAACCGTGAAAAGAT
	Reverse: GCAGGATGGCGTGAGGGAGAGCA
Dclk1	Forward: CAAGCCAGCCATGTCGTTC
	Reverse: TTCCTTTGAAGTAGCGGTCAC
IL-13	Forward: TGAGCAACATCACACAAGACC
	Reverse: GGCCTTGCGGTTACAGAGG
IL-25	Forward: ACAGGGACTTGAATCGGGTC
	Reverse: TGGTAAAGTGGGACGGAGTTG

Supplementary Table 2. Information of antibodies used in this study

Antibody	Catalog number	Vendor	Application	Dilution
Alexa Fluor® 555 Goat Anti-Mouse IgG (H+L)	A-21424	Invitrogen	IF	1:200
Alexa Fluor® 555 Goat Anti-Mouse IgG (H+L)	A-32727	Invitrogen	IF	1:200
Alexa Fluor® 488 Goat Anti-Rabbit IgG (H+L)	A-11070	Invitrogen	IF	1:200
Alexa Fluor® 488 DCLMK1	ab202754	Abcam	IF, PLA assay	1:200, 1:100
Alexa Fluor® 488 Lysozyme	NBP2-61118AF488	Novus	IF	1:200
beta actin; Sigma Aldrich	A5441	Sigma Aldrich	WB	1:10000
CD11b APC, clone M1/70	101212	BioLegend	Flow cytometry	1:50
CD16/32; BioLegend	101330	BioLegend	Flow cytometry	1:100
CD326 (EpCAM), clone G8.8	118202	BioLegend	Flow cytometry	1:200
CD4 APC, clone RM4-5	100516	BioLegend	Flow cytometry	1:50
CD45 FITC, clone 30-F11	103108	BioLegend	Flow cytometry	1:100
CD8 APC, clone 53-6.7	100712	BioLegend	Flow cytometry	1:50
DCLK1	ab88484	Abcam	IHC, IF	1:400
DCLK1	ab109029	Abcam	IHC	1:200
IL17Rb PE	12-7631-82	Thermo Fischer	Flow cytometry	1:50
IL-4 capture antibody	14-7041-85	ebioscience	ELISA	1:500
IL-4 biotin-labeled antibody	13-7042-85	ebioscience	ELISA	1:1000
IL-7Ra CF594, clone A7R34	135032	BioLegend	Flow cytometry	1:50
ITPR2	NB100-2466	Novus	PLA assay	1:50
KLRG1 PE/Cy7, clone 2F1/KLRG1	138416	BioLegend	Flow cytometry	1:50
Lrmp	orb166443	Biorbyt	IF, PLA assay, WB	1:200, 1:50, 1:1000
Mdm2(SMP14)	Sc-965	Santa Cruz	IF	1:200
MHC II (I-A/I-E) PE/Cy7, clone M5/114.15.2	107630	BioLegend	Flow cytometry	1:50
Mouse lineage antibody cocktail APC	558074	BD	Flow cytometry	5µl/sample
NK1.1 APC, clone	108710	BioLegend	Flow cytometry	1:50
p21(F-5)	sc-6246	Santa Cruz	IF	1:200
p53(FL-393)	sc-6243	Santa Cruz	ChIP	1:25
p53(CM5)	P53-PROTEIN-CM5	Leica Biosystems	IF	1:1000
Siglec-F PE, clone E50-2440	552126	BD	flow cytometry	1:50
