# Supplementary material for: Tumor suppressor p53 regulates intestinal type 2 immunity
Source: Nat Commun. 2021 Jun 7;12:3371. doi: 10.1038/s41467-021-23587-x (PMC8184793; doi:10.1038/s41467-021-23587-x)
Supplement: Supplementary file 3 — Reporting Summary [file 41467_2021_23587_MOESM3_ESM.pdf]

nature research

Corresponding author(s): Zhaohui Feng, Wenwei Hu

Last updated by author(s): Apr 1, 2021

Reporting Summary

Nature Research wishes to improve the reproducibility of the work that we publish. This form provides structure for consistency and transparency in reporting. For further information on Nature Research policies, see our Editorial Policies and the Editorial Policy Checklist.

Statistics

For all statistical analyses, confirm that the following items are present in the figure legend, table legend, main text, or Methods section.

n/a	Confirmed	
☐	☒	The exact sample size (*n*) for each experimental group/condition, given as a discrete number and unit of measurement
☐	☒	A statement on whether measurements were taken from distinct samples or whether the same sample was measured repeatedly
☐	☒	The statistical test(s) used AND whether they are one- or two-sided<br>*Only common tests should be described solely by name; describe more complex techniques in the Methods section.*
☒	☐	A description of all covariates tested
☒	☐	A description of any assumptions or corrections, such as tests of normality and adjustment for multiple comparisons
☐	☒	A full description of the statistical parameters including central tendency (e.g. means) or other basic estimates (e.g. regression coefficient) AND variation (e.g. standard deviation) or associated estimates of uncertainty (e.g. confidence intervals)
☒	☐	For null hypothesis testing, the test statistic (e.g. *F*, *t*, *r*) with confidence intervals, effect sizes, degrees of freedom and *P* value noted<br>*Give P values as exact values whenever suitable.*
☒	☐	For Bayesian analysis, information on the choice of priors and Markov chain Monte Carlo settings
☒	☐	For hierarchical and complex designs, identification of the appropriate level for tests and full reporting of outcomes
☒	☐	Estimates of effect sizes (e.g. Cohen's *d*, Pearson's *r*), indicating how they were calculated

Our web collection on statistics for biologists contains articles on many of the points above.

Software and code

Policy information about availability of computer code

Data collection	ILC-2 and Eosinophils were analyzed by Beckman-Coulter Cytomics FC500 Flow Cytometer.<br>Tuft cells were sorted by BD High Speed Cell Sorter.<br>Images of IF staining, organoid experiments, and PLAs assays were acquired using Nikon A1R-Si Confocal Microscope System.
Data analysis	The flow results were analyzed by FlowJo 10 software (Tree Star).<br>Quantification of tuft cell, goblet cell area and IF staining results was analyzed by image J software.<br>GraphPad Prism 8 was used for all the graphs and statistical analysis in this study.

For manuscripts utilizing custom algorithms or software that are central to the research but not yet described in published literature, software must be made available to editors and reviewers. We strongly encourage code deposition in a community repository (e.g. GitHub). See the Nature Research guidelines for submitting code & software for further information.

Data

Policy information about availability of data

All manuscripts must include a data availability statement. This statement should provide the following information, where applicable:

- Accession codes, unique identifiers, or web links for publicly available datasets
- A list of figures that have associated raw data
- A description of any restrictions on data availability

The data supporting the findings of this study are available within the paper and its supplementary Information files. The source data underlying Figs. 1b-1i, 2a-2g, 5a-5e, 5g-5i, 6a-6d are provided as Source data file. All other data are available from the corresponding author on reasonable request.

Field-specific reporting

Please select the one below that is the best fit for your research. If you are not sure, read the appropriate sections before making your selection.

☒ Life sciences ☐ Behavioural & social sciences ☐ Ecological, evolutionary & environmental sciences

For a reference copy of the document with all sections, see nature.com/documents/nr-reporting-summary-flat.pdf

Life sciences study design

All studies must disclose on these points even when the disclosure is negative.

Sample size: To analyze the levels of tuft cells, goblet cell area, ILC2, eosinophils, IL-13 mRNA levels, IL-25 mRNA levels, IL-4 and IL-25 levels, 5-8 mice/group were collected and analyzed.

Data exclusions: No data were excluded from this study.

Replication: Attempts to replicate the experiments have been performed successfully.
For animal experiments, the replication numbers (n=5-8) were described in the each figure. The age- and gender-matched mice were used for the replication experiments.
For Calcium experiment, at least a number of 90 cells were analyzed in each group.

Randomization: For animal experiments, all the age- and gender-matched mice were allocated into control or treatment groups randomly in this study.
For in vitro experiments, MEF cells from different genotypes were seeded in plates and randomly treated with different stimuli with clear label for further analysis(Including ChIP, WB, Calcium flux imaging, luciferase activity assays, RT-PCR assays)

Blinding: For animal experiment, different genotypes of mice were numbered and the investigators were blinded during data collection and analysis in this study.
For in vitro experiments, Val, 10(1) or MEF cells and the treatment were labeled clearly, so the experiments were not performed in a blinding manner.

Reporting for specific materials, systems and methods

We require information from authors about some types of materials, experimental systems and methods used in many studies. Here, indicate whether each material, system or method listed is relevant to your study. If you are not sure if a list item applies to your research, read the appropriate section before selecting a response.

Materials & experimental systems

n/a	Involved in the study	
☐	☒	Antibodies
☐	☒	Eukaryotic cell lines
☒	☐	Palaeontology and archaeology
☐	☒	Animals and other organisms
☒	☐	Human research participants
☒	☐	Clinical data
☒	☐	Dual use research of concern

Methods

n/a	Involved in the study	
☒	☐	ChIP-seq
☐	☒	Flow cytometry
☒	☐	MRI-based neuroimaging

Antibodies

Antibodies used:
Antibody-Vendor-Cat#-Research Resource Identifiers(RRID)
Alexa Fluor® 555 Goat Anti-Mouse IgG (H+L); Invitrogen Cat# A-21424; RRID:AB_141780
Alexa Fluor® 488 Goat Anti-Rabbit IgG (H+L); Invitrogen Cat# A-11070; RRID:AB_142134
Alexa Fluor® 488 DCLMK1; abcam Cat# ab202754
Alexa Fluor® 488 Lysozyme; Novus Cat# NBP2-61118AF488
beta actin; Sigma Aldrich Cat# A5441; RRID:AB_476744
CD11b APC, clone M1/70; BioLegend Cat# 101212;RRID:AB_312795
CD16/32; BioLegend Cat#101330
CD326 (EpCAM), clone G8.8; BioLegend Cat# 118202; RRID: AB_1089027
CD4 APC, clone RM4-5; BioLegend Cat# 100516;RRID:AB_312719
CD45 FITC, clone 30-F11; BioLegend Cat# 103108;RRID: AB_312973
CD8 APC, clone 53-6.7; BioLegend Cat# 100712;RRID:AB_312751
DCLK1; Abcam Cat# ab88484;RRID:AB_2041038
DCLK1; Abcam Cat# ab109029; RRID: AB_10864128

IL17Rb PE, clone MUNC33; Thermo Fischer Cat# 12-7361-82;RRID:AB_2572658
IL-4 capture antibody; ebioscience Cat#14-7041-85
IL-4 biotin-labeled antibody; ebioscience Cat#13-7042-85
IL-7Ra CF594, clone A7R34; BioLegend Cat# 135032;RRID:AB_2564217
ITPR2 Novus Cat# NB100-2466;RRID: AB_2280744
KLRG1 PE/Cy7, clone 2F1/KLRG1; BioLegend Cat# 138416;RRID: AB_2561736
Lrmp; biorbyt Cat# orb166443
Mdm2(SMP14) ; Santa Cruz Cat#Sc-965;RRID:AB_627920
MHC II (I-A/I-E) PE/Cy7, clone M5/114.15.2; BioLegend Cat# 107630;RRID:AB_2069376
Mouse lineage antibody cocktail APC; BD Cat# 558074;RRID:AB_1645213
NK1.1 APC, clone; BioLegend Cat# 108710;RRID:AB_313397
p21(F-5); Santa Cruz Cat#sc-6246;RRID:AB_628073
p53(FL-393); Santa Cruz Cat# SC-6243;RRID:AB_653753
p53(CM5); Leica Biosystems Cat# P53-PROTEIN-CM5; RRID:AB_563933
Siglec-F PE, clone E50-2440; BD Cat# 552126;RRID:AB_394341

The dilution ratio for each experiment was described in the Methods.

Validation

All the commercial antibodies were validated by the vendors and can be referenced to the data sheets listed on the websites.
For IF staining and WB experiments, proper positive and negative controls were included for validation.

Eukaryotic cell lines

Policy information about cell lines

Cell line source(s)

Mouse fibroblast Val5 and 10(1) cell lines are gifts from Dr. A. Levine at Institute for Advanced Study. H1299 cells were obtained from ATCC.
The WT, p53-/- and Lrmp-/- MEFs were freshly prepared from our lab and the detail procedure for MEF cells preparation was followed by the reference. (Harvey DM, Levine AJ. p53 alteration is a common event in the spontaneous immortalization of primary BALB/c murine embryo fibroblasts. Genes Dev 5, 2375-2385.)

Authentication

Val5 and 10(1) cells lines was confirmed through examination the p53 protein level by WB.
MEF cells from different genotypes of mice were confirmed by RT-PCR and WB after freshly made.

Mycoplasma contamination

All the cell lines in this study were test negative for mycoplasma contamination.

Commonly misidentified lines
(See ICLAC register)

N/A

Animals and other organisms

Policy information about studies involving animals; ARRIVE guidelines recommended for reporting animal research

Laboratory animals

WT and p53-/- (B6.129S2-Trp53tm1Tyj/J, stock number 002101) C57BL6/J mice were purchased from the Jackson Laboratory.
C57BL/6N-Lrmptm1a (EUCOMM)Wtsi/WtsiOulu (Lrmptm1a) mice were obtained from EUCOMM program.
Lrmp-/- mice were then back crossed with WT C57BL6/J for at least 5 generations after crossing with E2a-Cre mice (B6.FVB-Tg(E2a-cre)C5379Lmgd/J, The Jackson Laboratory, stock number 003724.

Wild animals

N/A

Field-collected samples

N/A

Ethics oversight

All animal procedures were approved by the Institutional Animal Care and Use Committee of Rutgers University.

Note that full information on the approval of the study protocol must also be provided in the manuscript.

Flow Cytometry

Plots

Confirm that:

- [x] The axis labels state the marker and fluorochrome used (e.g. CD4-FITC).
- [x] The axis scales are clearly visible. Include numbers along axes only for bottom left plot of group (a 'group' is an analysis of identical markers).
- [x] All plots are contour plots with outliers or pseudocolor plots.
- [x] A numerical value for number of cells or percentage (with statistics) is provided.

Methodology

Sample preparation	EOSI and ILC2 were isolated from suspension lamina propria cells using Lamina Propria Dissociation Kit (Miltenyi, Cat# 130-097-410) and gentleMACS™ Dissociator (Miltenyi, Cat# 130-093-235)
Instrument	Flow cytometry anlysis is performed by Beckman-Coulter Cytomics FC500 Flow Cytometer.
Software	Flow cytometry results were analyzed by FlowJo 10 software (Tree Star)
Cell population abundance	PI- CD45+ MHC II(I-A/I-E)- CD11b+ Siglec F+ cell populations were gated as eosinophils. CD45+ Lin-/low CD4- CD8- NK1.1- IL-7Ra+ KLRG1+ IL17Rb+ cell populations were gated as ILC2s.
Gating strategy	The gating strategies for ILC2 and eosinophils are provided in Fig S11.

☒ Tick this box to confirm that a figure exemplifying the gating strategy is provided in the Supplementary Information.
