# Supplementary material for: Tumor suppressor p53 regulates intestinal type 2 immunity
Source: Nat Commun. 2021 Jun 7;12:3371. doi: 10.1038/s41467-021-23587-x (PMC8184793; doi:10.1038/s41467-021-23587-x)

Figure1 Source data

| p53 | +/+ | | -/- | | p53 | +/+ | | -/- | |
|---|---|---|---|---|---|---|---|---|---|
| Tm | - | + | - | + | Tm | - | + | - | + |
| 1b-Tuft | 0.62 | 4.94 | 0.45 | 1.31 | 1c-Goblet | 50.55 | 152.14 | 52.80 | 58.73 |
| | 0.40 | 4.29 | 0.28 | 1.21 | | 44.34 | 129.44 | 53.32 | 74.20 |
| | 0.59 | 3.91 | 0.61 | 1.36 | | 51.40 | 96.56 | 52.03 | 62.21 |
| | 0.28 | 7.22 | 0.32 | 1.55 | | 64.39 | 96.83 | 47.00 | 102.01 |
| | 0.38 | 2.92 | 0.09 | 1.70 | | 45.12 | 103.81 | 55.46 | 67.80 |
| | 0.23 | 4.32 | 0.04 | 2.00 | | 47.55 | 126.05 | 50.93 | 61.48 |
| p53 | +/+ | | -/- | | p53 | +/+ | | -/- | |
| Tm | - | + | - | + | Tm | - | + | - | + |
| 1d-EOSI | 1.19 | 15.25 | 1.14 | 1.22 | 1d-ILC2 | 0.46 | 1.64 | 0.65 | 0.06 |
| | 1.47 | 10.92 | 0.57 | 2.78 | | 0.35 | 2.16 | 0.39 | 0.72 |
| | 2.06 | 10.82 | 3.98 | 4.81 | | 0.81 | 2.14 | 0.25 | 0.43 |
| | 3.37 | 13.02 | 2.30 | 2.92 | | 0.33 | 1.03 | 0.44 | 0.45 |
| | 3.79 | 18.99 | 0.51 | 5.04 | | 0.36 | 1.22 | 0.46 | 1.05 |
| | | 9.46 | | 5.69 | | | 2.64 | | 0.56 |
| p53 | +/+ | | -/- | | p53 | +/+ | | -/- | |
| Nb | - | + | - | + | Nb | - | + | - | + |
| 1e-IL-13 | 1.00 | 32.92 | 0.00 | 20.40 | 1g-Tuft | 0.50 | 6.67 | 0.34 | 1 |
| | 2.80 | 12.75 | 0.00 | 16.99 | | 0.39 | 6.07 | 0.46 | 3.61 |
| | 0.00 | 27.74 | 5.78 | 17.84 | | 0.42 | 7.46 | 0.50 | 0.48 |
| | 0.00 | 21.14 | 1.78 | 3.03 | | 0.97 | 5.17 | 0.85 | 0.96 |
| | 0.00 | 29.81 | 3.72 | 19.51 | | 0.70 | 7.88 | 0.31 | 4.31 |
| | | 29.59 | | 10.44 | | 0.33 | 4.08 | 0.55 | 1.55 |
| p53 | +/+ | | -/- | | p53 | +/+ | | -/- | |
| Nb | - | + | - | + | Nb | - | + | - | + |
| 1h-Goblet | 51.84 | 176.12 | 53.67 | 123.17 | 1i-EOSI | 3.71 | 35.26 | 1.50 | 10.07 |
| | 55.28 | 204.91 | 46.34 | 120.85 | | 3.26 | 38.99 | 3.59 | 7.55 |
| | 36.87 | 188.16 | 44.40 | 61.93 | | 2.17 | 15.97 | 3.08 | 13.95 |
| | 46.31 | 171.84 | 35.34 | 72.79 | | 3.61 | 25.69 | 1.85 | 3.62 |
| | 40.22 | 182.60 | 38.57 | 91.06 | | 3.56 | 9.41 | 1.61 | 3.19 |
| | 38.04 | 185.39 | 41.51 | 85.06 | | | 9.03 | | 4.35 |
| | | | | 79.36 | | | 14.87 | | 2.29 |
| p53 | +/+ | | -/- | | p53 | +/+ | | -/- | |
| Nb | - | + | - | + | Nb | - | + | - | + |
| 1i-ILC2 | 0.33 | 4.46 | 0.65 | 1.19 | 1j-IL-13 | 1.00 | 25.30 | 0.20 | 0 |
| | 0.67 | 2.80 | 0.39 | 1.31 | | 1.04 | 57.92 | 0.76 | 46.17 |
| | 0.36 | 2.50 | 0.25 | 1.30 | | 1.15 | 93.31 | 0.13 | 5.82 |
| | 0.40 | 1.72 | 0.44 | 0.84 | | 0.00 | 71.06 | 0.00 | 2.90 |
| | 0.35 | 1.84 | 0.46 | 1.30 | | 0.00 | 104.98 | 0.00 | 45.24 |
| | | | | 1.00 | | 0.00 | 89.82 | 0.26 | |

| Figure 2 Source data | | | | | | | | | |
|---|---|---|---|---|---|---|---|---|---|
| p53 | +/+ | | -/- | | p53 | +/+ | | -/- | |
| Sc | - | + | - | + | Sc | - | + | - | + |
| 2a-Tuft | 0.50 | 5.93 | 0.69 | 2.56 | 2a-Goblet | 54.84 | 150.61 | 60.81 | 71.51 |
| | 0.66 | 7.16 | 0.59 | 2.13 | | 49.87 | 171.42 | 58.79 | 89.92 |
| | 0.50 | 5.73 | 0.85 | 2.29 | | 48.47 | 208.60 | 59.12 | 124.40 |
| | 1.32 | 6.27 | 0.94 | 2.92 | | 56.56 | 225.47 | 40.95 | 122.89 |
| | 0.73 | 5.88 | 0.82 | 0.45 | | 49.00 | 171.54 | 42.00 | 50.88 |
| | 0.58 | 6.46 | 0.09 | 0.83 | | 57.82 | 190.13 | 51.07 | 54.87 |
| | | | | 0.57 | | | | | 52.63 |
| | | | | 0.82 | | | | | 43.97 |
| p53 | +/+ | | -/- | | p53 | +/+ | | -/- | |
| Sc | - | + | - | + | Sc | - | + | - | + |
| 2b-EOSI | 5.39 | 22.78 | 3.00 | 6.29 | 2b-ILC2 | 0.63 | 3.21 | 0.59 | 2.19 |
| | 3.13 | 15.73 | 3.98 | 9.15 | | 0.68 | 9.52 | 0.30 | 3.73 |
| | 2.84 | 10.52 | 1.89 | 7.86 | | 1.09 | 5.78 | 0.64 | 0.49 |
| | 1.69 | 7.24 | 3.13 | 4.08 | | 0.29 | 4.65 | 0.64 | 3.27 |
| | 2.96 | 11.20 | 2.06 | 5.50 | | 0.87 | 3.68 | 1.53 | 2.89 |
| | | 8.06 | | 4.28 | | | 6.68 | | 1.61 |
| p53 | +/+ | | -/- | | p53 | +/+ | | -/- | |
| Sc | - | + | - | + | IL-25 | - | + | - | + |
| 2c-IL-13 | 1.00 | 135.75 | 0.00 | 20.48 | 2e-Tuft | 0.32 | 3.81 | 0.51 | 5.82 |
| | 0.95 | 98.24 | 0.00 | 1.20 | | 0.64 | 7.95 | 0.55 | 6.50 |
| | 4.13 | 76.73 | 4.90 | 0.00 | | 0.52 | 5.26 | 0.99 | 5.81 |
| | 1.54 | 24.62 | 1.45 | 0.00 | | 0.41 | 6.43 | 0.62 | 5.80 |
| | 2.50 | 84.30 | 2.79 | 1.35 | | 0.29 | 5.09 | 0.24 | 6.46 |
| | | 46.88 | | | | 0.34 | 6.62 | 0.06 | 5.31 |
| p53 | +/+ | | -/- | | p53 | +/+ | | -/- | |
| IL-25 | - | + | - | + | IL-25 | - | + | - | + |
| 2e-Goblet | 48.34 | 91.50 | 52.58 | 154.18 | 2f-EOSI | 4.47 | 6.14 | 0.65 | 17.02 |
| | 42.26 | 204.43 | 66.90 | 120.00 | | 2.67 | 11.88 | 1.65 | 13.94 |
| | 36.95 | 158.27 | 41.69 | 147.51 | | 2.18 | 17.16 | 4.52 | 7.12 |
| | 42.95 | 180.95 | 57.11 | 165.55 | | 2.24 | 6.38 | 3.11 | 17.48 |
| | 62.75 | 160.97 | 43.54 | 147.47 | | 1.83 | 10.30 | 1.63 | 20.13 |
| | 57.82 | 162.96 | 43.53 | 140.37 | | 2.76 | 12.58 | 0.69 | 16.58 |
| p53 | +/+ | | -/- | | p53 | +/+ | | -/- | |
| IL-25 | - | + | - | + | IL-25 | - | + | - | + |
| 2f-ILC2 | 0.76 | 2.66 | 0.84 | 4.32 | 2g-IL-13 | 1.00 | 40.55 | 0.00 | 32.36 |
| | 0.93 | 4.07 | 1.15 | 4.33 | | 0.00 | 51.86 | 0.00 | 12.12 |
| | 0.91 | 4.66 | 0.68 | 2.21 | | 2.62 | 57.43 | 0.00 | 11.42 |
| | 0.54 | 5.32 | 0.61 | 6.03 | | 0.00 | 34.48 | 2.15 | 32.44 |
| | 0.59 | 5.50 | 0.71 | 2.44 | | 2.13 | 14.80 | 8.70 | 22.26 |
| | | 3.98 | | 2.69 | | | 16.99 | | |
| p53 | +/+ | | | | -/- | | | | |
| Treatment | Con | Tm | Nb | Sc | Con | Tm | Nb | Sc | |
| 2d-IL-25 | 1.00 | 26.50 | 143.71 | 92.41 | 2.25 | 1.21 | 0.30 | 0.27 | |
| | 2.66 | 85.15 | 55.02 | 33.85 | 0.64 | 9.65 | 8.49 | 0.59 | |
| | 1.85 | 32.90 | 134.74 | 30.40 | 0.10 | 1.22 | 3.89 | 0.34 | |
| | 0.64 | 111.05 | 1016 | 110.89 | 1.75 | 1.21 | 3.12 | 3.40 | |
| | 0.39 | 90.20 | 617.80 | 92.41 | 1.42 | 10.97 | 1.34 | 4.49 | |
| | 0.50 | 69.45 | 28.84 | | 0.08 | 8.50 | 55.06 | | |
| | 0.50 | | | | 0.33 | | 21.80 | | |
| | | | | | | | 54.19 | | |

Figure 5 Source data

| Lrmp | +/+ | | -/- | | Lrmp | +/+ | | -/- | |
|---|---|---|---|---|---|---|---|---|---|
| Tm | - | + | - | + | Nb | - | + | - | + |
| 5a-Tuft | 0.24 | 5.02 | 0.30 | 0.78 | 5a-Tuft | 0.37 | 5.54 | 0.83 | 0.59 |
| | 0.27 | 3.00 | 0.41 | 0.76 | | 0.28 | 5.38 | 0.58 | 0.60 |
| | 0.28 | 2.95 | 0.22 | 0.16 | | 0.53 | 7.46 | 0.60 | 1.55 |
| | 0.37 | 6.59 | 0.45 | 0.39 | | 0.50 | 4.91 | 0.32 | 0.70 |
| | 0.43 | 2.82 | 0.30 | 0.67 | | 0.33 | 8.12 | 0.43 | 1.28 |
| | 0.21 | 4.79 | 0.39 | 0.51 | | 0.39 | 4.11 | 0.48 | 1.16 |
| Lrmp | +/+ | | -/- | | Lrmp | +/+ | | -/- | |
| Tm | - | + | - | + | Nb | - | + | - | + |
| 5b-Goblet | 50.62 | 149.74 | 53.97 | 45.56 | 5b-Goblet | 51.37 | 219.81 | 56.06 | 64.83 |
| | 50.86 | 115.18 | 45.64 | 59.65 | | 44.58 | 178.35 | 52.23 | 56.21 |
| | 61.56 | 109.33 | 45.01 | 54.80 | | 51.35 | 180.63 | 50.31 | 62.94 |
| | 54.68 | 121.36 | 46.99 | 69.57 | | 45.75 | 145.67 | 47.54 | 91.98 |
| | 67.51 | 131.03 | 58.95 | 80.72 | | 40.44 | 182.23 | 47.45 | 105.22 |
| | 51.60 | 168.95 | 48.06 | 62.20 | | 44.85 | 160.91 | 37.83 | 73.33 |
| Lrmp | +/+ | | -/- | | Lrmp | +/+ | | -/- | |
| Tm | - | + | - | + | Nb | - | + | - | + |
| 5c-EOSI | 2.37 | 13.05 | 4.70 | 2.44 | 5c-EOSI | 3.77 | 12.26 | 1.95 | 7.40 |
| | 1.35 | 8.75 | 1.98 | 4.03 | | 3.27 | 10.82 | 3.82 | 4.04 |
| | 1.72 | 9.34 | 2.55 | 4.92 | | 2.45 | 16.74 | 3.56 | 5.83 |
| | 2.60 | 18.99 | 2.80 | 3.02 | | 3.48 | 36.38 | 2.75 | 8.25 |
| | 3.20 | 5.66 | 2.44 | 4.60 | | 4.45 | 12.90 | 2.69 | 7.37 |
| | | 9.57 | | 4.59 | | | 22.71 | | 3.45 |
| Lrmp | +/+ | | -/- | | Lrmp | +/+ | | -/- | |
| Tm | - | + | - | + | Nb | - | + | - | + |
| 5d-ILC2 | 0.55 | 1.63 | 0.48 | 0.77 | 5d-ILC2 | 0.55 | 3.80 | 0.67 | 0.83 |
| | 1.01 | 2.21 | 1.02 | 0.53 | | 0.46 | 2.38 | 0.49 | 1.31 |
| | 0.25 | 1.91 | 0.84 | 1.21 | | 0.25 | 2.66 | 0.31 | 0.69 |
| | 0.35 | 2.62 | 1.01 | 1.05 | | 0.35 | 1.81 | 0.48 | 0.73 |
| | 0.49 | 1.27 | 0.59 | 0.62 | | 0.49 | 1.94 | 0.54 | 0.36 |
| | | 2.37 | | 0.82 | | | | | 0.69 |
| Lrmp | +/+ | | -/- | | Lrmp | +/+ | | -/- | |
| Tm | - | + | - | + | Nb | - | + | - | + |
| 5e-IL-13 | 0.00 | 23.08 | 0.00 | 1.18 | 5e-IL-13 | 0.00 | 27.74 | 0.00 | 3.19 |
| | 1.05 | 34.20 | 0.59 | 3.92 | | 0.00 | 24.99 | 0.58 | 0.33 |
| | 2.17 | 29.37 | 1.66 | 4.64 | | 0.00 | 62.64 | 1.10 | 1.74 |
| | 0.00 | 28.96 | 0.00 | 3.34 | | 0.00 | 42.43 | 0.38 | 0.58 |
| | 0.00 | 11.24 | 5.23 | 0.00 | | 0.62 | 65.48 | 0.54 | 0.67 |
| | | 33.52 | | 0.00 | | 0.00 | 82.05 | 0.25 | |
| | | | | 2.12 | | | | 0.00 | |
| | | | | 3.92 | | | | | |
| | | | | 4.64 | | | | | |
| Lrmp | +/+ | | -/- | | Lrmp | +/+ | | -/- | |
| Sc | - | + | - | + | Sc | - | + | - | + |
| 5g-Tuft | 0.85 | 6.43 | 0.35 | 2.16 | 5g-Goblet | 55.22 | 145.61 | 60.01 | 74.07 |
| | 0.68 | 7.22 | 0.32 | 2.09 | | 59.74 | 178.94 | 59.19 | 87.10 |
| | 0.44 | 5.54 | 0.56 | 0.96 | | 54.11 | 205.64 | 47.58 | 78.88 |
| | 0.34 | 7.42 | 0.71 | 0.96 | | 48.92 | 261.45 | 55.43 | 58.56 |
| | 0.37 | 5.51 | 1.07 | 1.20 | | 55.70 | 193.95 | 57.72 | 45.28 |
| | | 8.35 | | 0.29 | | | 177.78 | | 48.94 |
| | | | | 0.54 | | | | | 51.92 |
| | | | | 0.76 | | | | | 55.67 |
| Lrmp | +/+ | | -/- | | Lrmp | +/+ | | -/- | |
| Sc | - | + | - | + | Sc | - | + | - | + |
| 5h-EOSI | 2.08 | 17.59 | 2.55 | 6.29 | 5h-ILC2 | 0.65 | 5.07 | 0.61 | 1.05 |
| | 4.04 | 28.92 | 2.07 | 4.28 | | 1.85 | 9.52 | 1.63 | 1.34 |
| | 2.82 | 10.08 | 3.12 | 4.15 | | 1.42 | 8.46 | 0.91 | 1.75 |
| | 3.77 | 7.49 | 2.08 | 6.65 | | 0.58 | 4.84 | 0.64 | 0.51 |
| | 2.48 | 11.97 | 2.15 | 7.28 | | 0.86 | 3.85 | 0.68 | 1.40 |
| | | 8.06 | | 7.25 | | | 5.31 | 0.73 | 1.09 |
| | | | | 7.44 | | | | | 3.09 |
| Lrmp | +/+ | | -/- | | | | | | |
| Sc | - | + | - | + | | | | | |
| 5i-IL-13 | 1.12 | 84.46 | 0.00 | 0.00 | | | | | |
| | 0.53 | 110.17 | 0.40 | 0.00 | | | | | |
| | 0.00 | 92.21 | 0.00 | 1.99 | | | | | |
| | 3.44 | 34.50 | 0.58 | 0.00 | | | | | |
| | 0.00 | 52.39 | 1.54 | 0.00 | | | | | |
| | | 36.06 | | | | | | | |

Figure 6 Source data

| Lrmp | +/+ | | | | -/- | | | |
|---|---|---|---|---|---|---|---|---|
| Treatment | Con | Tm | Nb | Sc | Con | Tm | Nb | Sc |
| 6a-IL-25 | 1.00 | 22.50 | 126.59 | 81.97 | 1.16 | 2.24 | 10.92 | 1.94 |
| | 2.31 | 86.10 | 50.91 | 27.97 | 0.49 | 0.95 | 5.85 | 2.15 |
| | 2.31 | 31.19 | 136.24 | 27.21 | 1.30 | 0.68 | 5.55 | 9.15 |
| | 1.84 | 88.77 | 859 | 112.13 | 0.48 | 24.17 | 2.42 | 0.27 |
| | 0.51 | 25.16 | 692.18 | 81.97 | 2.14 | 1.18 | 11.77 | 3.86 |
| | 0.50 | 110.28 | | | 0.93 | 0.76 | 11.21 | 5.05 |
| | 0.75 | 117.30 | | | 0.29 | | | |
| | 0.34 | | | | | | | |
| | 0.80 | | | | | | | |

| Lrmp | +/+ | | -/- | |
|---|---|---|---|---|
| IL-25 | - | + | - | + |
| 6b-Tuft | 1.21 | 5.23 | 0.15 | 7.25 |
| | 0.39 | 7.95 | 0.10 | 8.83 |
| | 0.19 | 4.26 | 0.53 | 4.38 |
| | 0.14 | 3.84 | 0.23 | 6.76 |
| | 0.32 | 4.89 | 0.36 | 5.38 |
| | 0.74 | 5.66 | 1.38 | 6.51 |
| | | | | 7.30 |

| Lrmp | +/+ | | -/- | |
|---|---|---|---|---|
| IL-25 | - | + | - | + |
| 6b-Goblet | 39.20 | 90.11 | 66.20 | 95.49 |
| | 41.27 | 171.65 | 61.93 | 157.80 |
| | 46.29 | 141.50 | 65.57 | 137.95 |
| | 64.37 | 143.52 | 42.77 | 133.92 |
| | 44.30 | 150.48 | 50.06 | 160.17 |
| | 48.13 | 167.09 | 46.51 | 158.11 |
| | | | | 162.05 |

| Lrmp | +/+ | | -/- | |
|---|---|---|---|---|
| IL-25 | - | + | - | + |
| 6c-EOSI | 4.76 | 8.19 | 6.11 | 4.81 |
| | 3.91 | 12.24 | 1.56 | 4.37 |
| | 1.95 | 6.28 | 1.58 | 12.74 |
| | 1.94 | 9.83 | 3.47 | 13.28 |
| | 3.47 | 11.96 | 3.42 | 5.36 |
| | | 12.92 | | 12.43 |
| | | | | 11.22 |

| Lrmp | +/+ | | -/- | |
|---|---|---|---|---|
| IL-25 | - | + | - | + |
| 6c-ILC2 | 0.98 | 3.42 | 0.50 | 4.53 |
| | 0.95 | 9.98 | 1.69 | 9.78 |
| | 0.46 | 2.69 | 2.69 | 5.85 |
| | 0.79 | 2.74 | 0.56 | 2.96 |
| | 0.72 | 5.33 | 0.70 | 3.03 |
| | | 3.40 | | 4.35 |

| Lrmp | +/+ | | -/- | |
|---|---|---|---|---|
| IL-25 | - | + | - | + |
| 6d-IL-13 | 0.00 | 53.14 | 2.61 | 22.66 |
| | 0.00 | 34.43 | 1.43 | 10.63 |
| | 3.18 | 20.17 | 2.61 | 17.96 |
| | 3.21 | 60.62 | 2.47 | 26.09 |
| | 1.64 | 8.36 | 3.16 | 51.50 |
| | | 17.91 | | |

# Uncropped blots for results presented in the main figures

Figure 3g

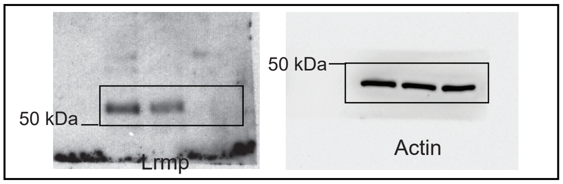


Figure 4c

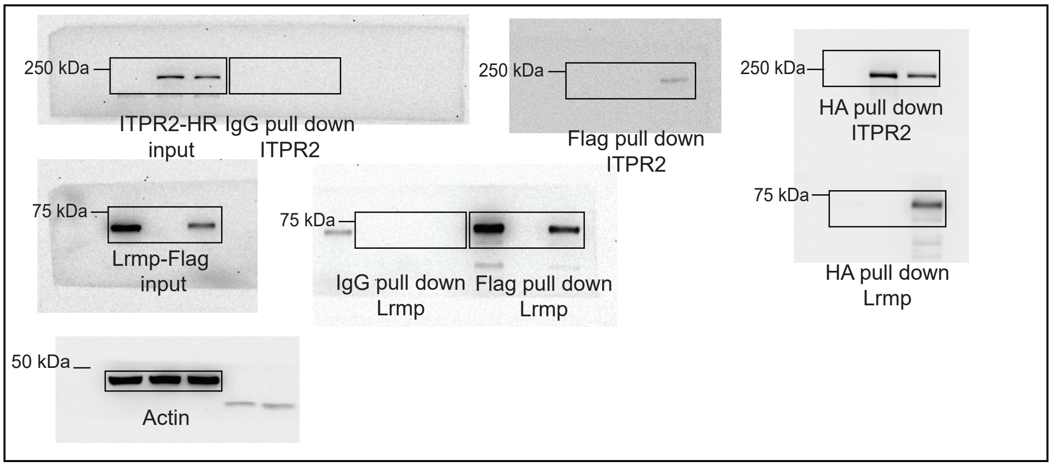


Figure 4d

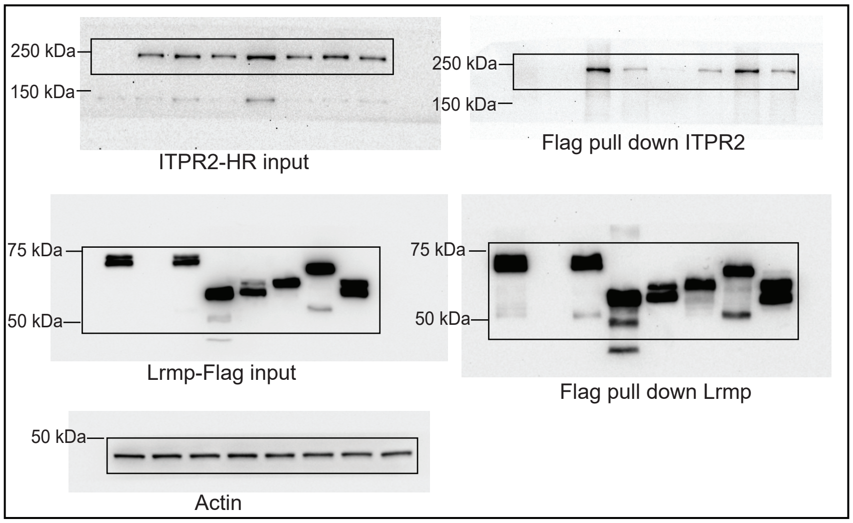

Supplement: Supplementary file 4 — Source Data [file 41467_2021_23587_MOESM4_ESM.pdf]
